# A Qualitative Evaluation of the Acceptability of a Tailored Smartphone Alcohol Intervention for a Military Population: Information About Drinking for Ex-Serving Personnel (InDEx) App

**DOI:** 10.2196/12267

**Published:** 2019-05-24

**Authors:** Jo-Anne Puddephatt, Daniel Leightley, Laura Palmer, Norman Jones, Toktam Mahmoodi, Colin Drummond, Roberto J Rona, Nicola T Fear, Matt Field, Laura Goodwin

**Affiliations:** 1 Department of Psychological Sciences Institute of Life and Human Sciences University of Liverpool Liverpool United Kingdom; 2 King's Centre for Military Health Research Institute of Psychiatry, Psychology & Neuroscience King's College London London United Kingdom; 3 Academic Department of Military Mental Health Institute of Psychiatry, Psychology & Neuroscience King's College London London United Kingdom; 4 Department of Informatics King's College London London United Kingdom; 5 Addictions Department Institute of Psychiatry, Psychology & Neuroscience King's College London London United Kingdom; 6 South London and Maudsley NHS Foundation Trust London United Kingdom; 7 Department of Psychology University of Sheffield Sheffield United Kingdom

**Keywords:** behavior, alcohol drinking, veterans, interview, smartphone

## Abstract

**Background:**

Alcohol consumption in the UK Armed Forces is higher than in the general population, and this pattern continues after leaving the service. Smartphone apps may be useful to increase ex-serving personnel’s awareness of their alcohol consumption, support self-monitoring, and prompt a change in behavior.

**Objective:**

The study aimed to explore the acceptability of *Information about Drinking in Ex-serving personnel* (InDEx), a tailored smartphone app, combined with personalized short message service (SMS) text messaging designed to target ex-serving personnel who meet the criteria for hazardous alcohol use.

**Methods:**

The InDEx intervention included 4 key modules: (1) assessment and normative feedback, (2) self-monitoring and feedback, (3) goal setting and review, and (4) personalized SMS text messaging. A semistructured telephone interview study was conducted with ex-serving personnel after using the app for a 28-day period. Interviews were used to explore the acceptability of app modules and its functionality and the perceived changes in participant’s drinking. Interview transcripts were analyzed using inductive thematic analysis.

**Results:**

Overall, 94% (29/31) participants who used InDEx agreed to take part in a telephone interview. Overall, 4 themes were identified: *Credibility*, *Meeting their needs*, *Simplicity*, and *Helpful for ex-serving personnel*. The importance of credibility, functionality, and meeting the individual needs of ex-serving personnel was emphasized. Acceptability and engagement with specific modules of the app and text messages were influenced by the following: (1) if they felt it provided credible information, (2) whether the content was appropriately personalized to them, (3) the ease of use, and (4) beliefs about their own drinking behaviors. Participants recommended that the app would be most suitable for personnel about to leave the Armed Forces.

**Conclusions:**

InDEx was an acceptable smartphone app for ex-serving personnel for monitoring alcohol consumption and in providing meaningful feedback to the individual. Pages that met the participant’s interests and provided real time personalized, credible feedback on their drinking and text messages tailored to participant’s interactions with the app were particularly favored.

## Introduction

### Background

The prevalence of hazardous drinking within the UK Armed Forces is higher than in the general population [1], and this pattern continues after personnel leave service [2]. There is little research on drinking motivations in military populations, but some evidence indicates drinking as a maladaptive coping response to psychological distress [3] and for social and physical pleasure [4]. Research has shown that there is a perceived stigma around reporting alcohol problems [5], which may be a barrier to face-to-face help seeking [6]. There are also issues around individuals recognizing their drinking being a problem [7], specifically in the military where approximately one-third of those who meet the criteria for harmful alcohol use recognize a problem [8]. Given the high prevalence of hazardous drinking within the Armed Forces and the potential for additional pressures when transitioning to civilian life, it is important to identify a suitable intervention that can target alcohol use in this population.

Web- and smartphone app–based interventions (eg, Down Your Drink [9], A-CHESS [10], DrinkLess [11], and LBMI-A [12]), through formative methods, have been harnessed to increase the reach and provide real-time monitoring and personalized delivery. Self-monitoring and real-time information about one’s drinking through the use of an app may help individuals identify if they have a problem.

Recent research indicates that smartphone alcohol app interventions may be effective for changing drinking behavior, for example, a systematic review of smartphone alcohol app interventions for adults found that 5 of the 6 studies reported significant changes in alcohol consumption [13]. Furthermore, digital alcohol interventions have been found to be as effective as face-to-face interventions [14]. Present alcohol apps have focused on self-monitoring, which is known to lead to behavior change [15,16], but a review of which behavior change techniques (BCTs) work in alcohol interventions has indicated that credible sources (communication from a credible source in favor of or against behaviors [17]) or suggesting alternative behaviors may also be effective techniques [16].

Although some alcohol apps have been shown to be effective, there are some issues to consider. One example is maintaining user engagement where research has shown that users engage less as the study progresses [18,19]. One possible way in which monitoring can be maintained is through the use of text messages encouraging adherence [20,21]. Another issue is the extent to which a theory is used to develop alcohol app interventions [22-24]; research has shown that more than half of digital alcohol interventions did not mention the use of theory [14,25] and only 38% used theory to inform intervention development [25]. Therefore, it is difficult to understand if it is effective in changing drinking behavior [14].

Given the prevalence of hazardous drinking in the Armed Forces and the potential for smartphone apps and text messages to target hard-to-reach populations and prompt changes in drinking behavior, we developed a theory-based intervention to target hazardous and harmful drinking in ex-serving military personnel which also incorporated BCTs: *Information about Drinking in Ex-serving personnel* (InDEx) [26,27]. InDEx is aimed at ex-serving personnel who are likely to be experiencing short-term consequences of their drinking but might not be aware of this being a problem. To the best of our knowledge, this is the first smartphone-based alcohol intervention that uses both an app and personalized and tailored text messaging.

We conducted a feasibility study with ex-serving personnel to explore adherence and engagement with InDEx over a 28-day period. Our usability findings have been published elsewhere [27], but briefly, we found good engagement (having at least 3 client-server interactions in a 7-day period) throughout this period, with 23 out of 31 participants using the app every week and 27 using the app in the final week. Self-monitoring and feedback modules were most frequently used compared with goal setting and normative feedback [27].

Although usability data from the app inform intervention developers of how participants engaged with certain features, it does not necessarily tell us why this occurred [28]. Using qualitative methods allows us to understand our usability findings further in addition to providing insight into the acceptability of the app for ex-serving personnel, particularly as this is the first of its kind to target alcohol use in this population. For the purpose of this study, we define acceptability in terms of how useful participants perceived InDEx for ex-serving personnel and whether it was useful for monitoring alcohol consumption in terms of positive and negative comments [29].

### Objectives

The aim of this study, therefore, was to (1) explore the acceptability of InDEx for an ex-serving population based on their experience of using the app over a 28-day period and (2) explore participant’s experience of using key modules to monitor their alcohol consumption using qualitative methods.

## Methods

Ethical approval was obtained from the local research ethics committee at the University of Liverpool (reference: #0625).

### Information About Drinking in Ex-Serving Personnel App

**Figure 1:**
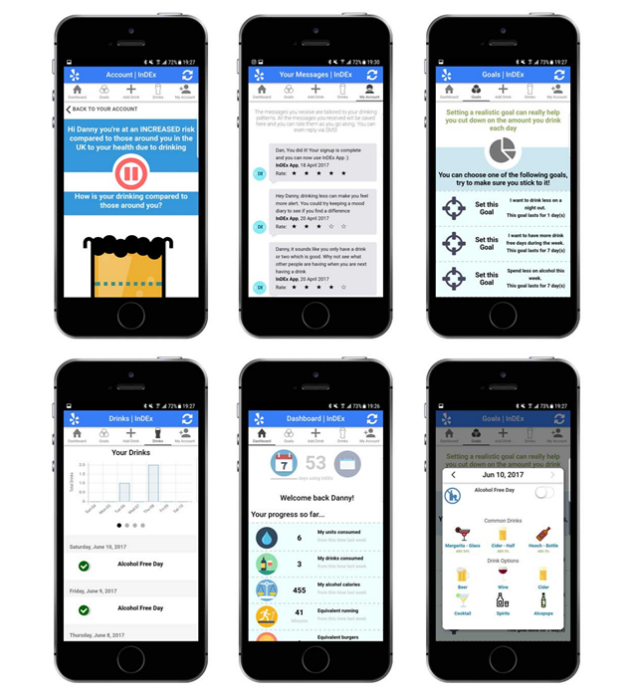
Example screenshots of interactions with the InDEx app. Top left to bottom right: normative feedback, personalized text message history, set a goal, drink diary, dashboard, and add a drink. InDEx: Information about Drinking in Ex-serving personnel.

InDEx used agile development methodologies with each cycle, focusing firstly on the development and secondly on stakeholder and expert user testing [26] (see Figure 1).

InDEx was targeted to ex-serving personnel who had recently left the military. The app was informed by the Health Action Process Approach [30] and social norms theory [31], where delivery was split into 3 stages: (1) normative feedback, action self-efficacy, and self-monitoring; (2) maintenance self-efficacy and action planning; and (3) recovery self-efficacy and coping planning. The app was complemented by personalized and tailored text messaging to provide prompts to log into the app, suggest alternative behaviors, and provide feedback on goals set [26,27]. A full description of InDEx, including its source code, message bank, and rules-based approach for tailoring, is available in the study by Leightley et al [26,27]. A summary is provided hereafter.

Upon consenting to take part in the feasibility study, participants were sent a link via email to download InDEx. Participants were provided with a navigation page to explain how to use each of the following modules:

1. Assessment and normative feedback: participants were provided an infographic representing participants’ self-reported alcohol consumption compared with the general population. This could be revisited at any time in the *How you compare* page under *My account*.
2. Self-monitoring and feedback: participants recorded their alcohol consumption using a drinks diary under the *Add drink* page from a list of predefined drinks; alcohol and calorie content and the price of a drink could be edited. Participants were provided feedback on their drinking under the *Dashboard* and *Drinks* pages.
3. Goal (setting and review): participants could set a goal based on implementation intentions [32] (ie, they identified the barrier and solution for each goal) using 1 of 4 options: (1) *I want to drink less on a night out*, (2) *I want to have more drink-free days during the week*, (3) *I want to spend less on alcohol this week*, or (4) *I will have a maximum of X drinks*. Goals set were viewed under the *Goals* page.
4. Personalized short message service (SMS) text messaging: a bank of 180 tailored and personalized text messages was developed and included BCTs found to be useful in alcohol interventions [16,22]. BCTs are defined as *active ingredients* of an intervention designed to change behavior [16,33,34]. The message bank and decision tree for sending text messages are available upon request from the corresponding author.

### Participants

Participants in the feasibility study were recruited from the King’s Centre for Military Health Research (KCMHR) Health and Wellbeing cohort [35-37], an ongoing study of UK Armed Forces personnel since 2003, including those who had left the military. Potential participants were selected from the cohort database based on meeting the following inclusion criteria: (1) were ex-serving personnel who left the Armed Forces within the last 2 years (at the time of sampling—May 2017); (2) aged 18 to 65 years; (3) who owned a smartphone; and (4) who had an Alcohol Use Disorder Identification Test (AUDIT) score of 8 to 19 at phase 2 of the cohort, which was conducted in 2009, indicating hazardous or harmful alcohol use according to the AUDIT. Those scoring above 20 on the AUDIT meet the criteria for probable alcohol dependency, and we felt that they may require more intensive treatment [27,38]. An additional criterion for the study was for participants to have taken part in the feasibility study [27].

For this study, purposive sampling was used to recruit all participants who had taken part in the feasibility study to ensure that we were able to capture all their experiences of using InDEx. Participants were invited to the interview using their email address after completing our feasibility study between June and August 2017. Participants were reimbursed £40 for using the app for a 28-day period.

#### Procedure

Participants were invited by JP, via email, to take part in a telephone interview up to 3 weeks after using InDEx for a 28-day period. If the participant did not respond to this initial email, a follow-up email and SMS text message was sent.

Semistructured telephone interviews were conducted by a trained postgraduate qualitative research assistant (JP); these were used to allow participants to discuss their experience of the app. No relationship was established between JP and participants before the study other than when inviting to the interview. Participants were aware that JP was a research assistant at the University of Liverpool and part of the InDEx app team. To minimize response bias, participants were briefed on the purpose of the study and were encouraged to be honest in their responses as it would be used to inform future intervention development.

The topic guide was developed by the authors and pilot-tested within the project team but did not influence interviews. Topics included the ease of use and accessibility of the app, thoughts about the content and frequency of the text messages, and perceptions about providing information about participants’ drinking. The interview also included questions around the suitability of InDEx for ex-serving personnel. The topic guide can be found in Multimedia Appendix 1. Follow-up questions were allowed, and participants were able to add any additional comments that had not been covered. Field notes were also made during the interviews to inform potential follow-up questions.

Participants were informed before the interview that their responses would be audio-recorded using a digital Dictaphone and transcripts would be pseudoanonymized and stored securely. No other persons were present during the interview. Telephone interviews took place a median of 8 days after completing the feasibility study (range 2 to 23) and were conducted between June and August 2017. Repeat interviews did not take place. The mean length of the interview was 30 min and 22 seconds (range: 16 min and 38 seconds to 39 min and 37 seconds).

#### Analysis

Interviews were audio-recorded and transcribed verbatim by a professional transcriptionist. Transcripts were checked with audio recordings to determine accuracy and were pseudoanonymized. Inductive thematic analysis was conducted to allow for the identification of patterns within data. This allows themes and codes to be strongly linked to the data, as opposed to being theory driven [39]. This involves a 5-phase process of familiarizing with the data, generating initial codes, searching for themes, reviewing themes, and finally defining and naming themes. This analytical method has been frequently used in previous studies aiming to explore the acceptability of smartphone health interventions [11,40,41].

Analysis followed an iterative process whereby themes were identified and merged or removed during the continual analysis of raw data. NVivo 10 was used to facilitate the coding process, and a subset of transcripts (N=3) were randomized and sent to a second coder (LP) along with a developed codebook to provide feedback on this codebook and code the transcripts and to establish reliability. JP reviewed the coded transcripts for coding consistency and any disagreements were reviewed by a third coder (LG). The codebook developed from our analysis is available upon request to the corresponding author.

We also triangulated our qualitative analysis with participants’ AUDIT scores, interactions with InDEx, and self-reported units consumed from week 1 to week 4 of the study to provide context to their interview transcripts (see Multimedia Appendix 2).

## Results

### Overview

A total of 29 participants (94%, 29/31) agreed to be interviewed, of whom 25 (85%, 25/29) were male. Furthermore, 18 participants (62%, 18/29) served in the Army, 6 (21%, 6/29) in the Royal Air Force, and 5 (17%, 5/29) in the Navy. In addition, 23 participants (79%, 23/29) served as a noncommissioned officer or other rank, and 24 participants (83%, 24/29) served in the Armed Forces for more than 12 years. Moreover, 2 participants invited to take part in the interview did not respond to emails; therefore, it is not known why they did not wish to participate.

At baseline, participants reported a median AUDIT score of 11 (range 5 to 16), suggesting that some participants’ AUDIT scores had decreased since taking part in the KCMHR Health and Wellbeing cohort (see Multimedia Appendix 2). Overall, 10 (35%, 10/29) participants reported using a health app previously, but no participants previously used an alcohol app. The median number of interactions (see Multimedia Appendix 2) reflects how frequently participants recorded a drink, completed questionnaires, and rated text messages.

### Overall Themes

**Figure 2:**
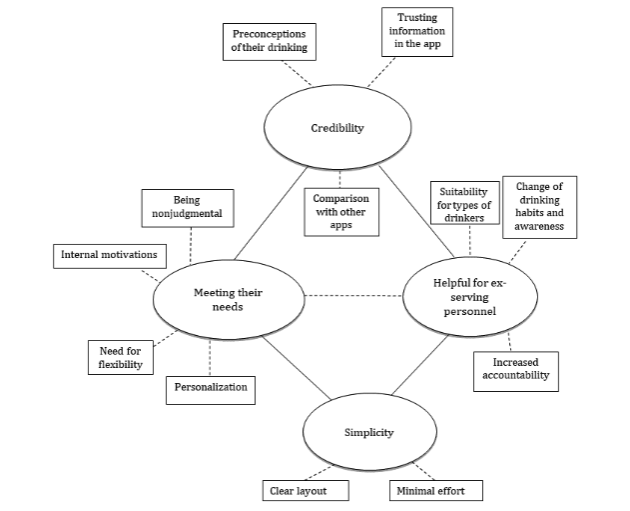
Thematic map overview of themes and subthemes.

Overall, the InDEx app was acceptable for ex-serving personnel to monitor their alcohol consumption and understand their drinking habits based on positive participant feedback. A total of 4 overarching themes were developed: (1) *Credibility*, (2) *Meeting their needs*, (3) *Simplicity*, and (4) *Helpful for ex-serving personnel* (Figure 2).

#### Theme 1: Credibility

The InDEx app was generally considered as a credible tool for participants; however, some features were deemed more credible than others, for example, the *Dashboard* page compared with the *How you compare* page. The theme of credibility hinged upon the 3 subthemes of preconceptions of their drinking, comparison with other apps, and trusting information in the app. These were interlinked as participants referenced their own drinking and experience of using other apps to determine how credible the app and its specific features were. These judgements influenced whether participants engaged with the app and whether they believed the information provided, subsequently impacting perceived changes in accountability of their drinking behavior. Perceived credibility was therefore highly influential in determining the acceptability of InDEx and level of engagement.

##### Comparison With Other Apps

As participants had not previously used an alcohol app, they drew from their experiences using other health and fitness apps, for example, Fitbit, to develop recommendations of how to enhance the acceptability and credibility of InDEx:

I suppose it would be a bit more usable if there were (umm) a way to (umm) enter a...like a make or a (umm) a label scanner or something like that so that (umm) it was a bit more accurate.

P3, male

Some participants felt the app could include additional features, such as recording their diet, in accordance with other health apps, to provide an accurate reflection of the contexts of their drinking:

...a text message came through saying that...basically says eat whilst you’re drinking...Yet on the App, ...you could add a drink (umm) you could say where you were, were you with anybody, but then you couldn’t say whether you were eating...where I think that might be useful because...if you’re promoting try eating when you drink as a means to reduce your consumption, it would be nice to know that you were actually out for a meal...I could then when I look at it...on the day-to-day drinking. So yeah I was out for a meal then, we had quite a lot to drink in effect, oh but we were out and we had a big meal or that at a restaurant. Just as an indication, because then it gives you...reasons why that...that consumption was higher that day than any other day sort of thing.

P10, male

Expectations derived from other app experiences therefore informed perceptions of InDEx’s credibility and could impact participants’ engagement with the app as a method for monitoring their alcohol consumption.

##### Preconceptions of Their Drinking

Not all participants perceived themselves as a problem drinker, neither did they use recommended drinking guidelines to inform their drinking. Instead, participants relied upon their own perceptions of both their personal drinking habits and those of others to determine the levels of risk. The below quote is from a participant with an AUDIT score of 14 who did not classify themselves as a problem drinker and seemed to attribute this to their drinking pattern:

Because again (sigh) I don’t see myself as a...as a big drinker. So I...I’m not really doing more than twenty units in a week and I tend not to drink too much during the week...

P21, male

This belief appeared to influence the perceptions of whether the information participants received about their alcohol consumption was credible, in particular, the *How you compare* page, where participants assumed that they drank less than their peers and, therefore, questioned the credibility of this comparison information:

I class myself as a moderate drinker, even though I think the App reckons I do drink too much. (umm) But compared (laugh) to an awful lot of people I know, I don’t.

P2, male

I think it (how you compare page) said that...I was higher than (sigh)...It said I was like (umm) the 60% of the population drinking and stuff.

P21, male

What did you think about that information that you were given?

Interviewer

(umm) I think that the population are good liars! (laugh)

P21, male

##### Trusting Information in the App

There were mixed opinions of how credible different pages of InDEx were and some pages appeared more credible than others:

I’m not too sure because (umm) it (how you compare page) said that 20% of the people in the country drink less than me! (laugh) And I find that quite hard! (laugh)...because I don’t drink a lot! So (umm) in the time I’ve been using the App I’ve...I’ve probably only been drinking on about four occasions...in the month. So...(umm) to say that 20% of people in the country, that probably means people who don’t drink!

P20, male

I’ve just got into the dashboard...seven days using it, (umm) bring up a new total, it knows my name and my progress. (umm) See because I’ve had a breather, everything is down to zero this week

P6, male

Participants also questioned where information about their drinking came from, particularly with the *How you compare* page, and the information was considered less credible when the source of information was not apparent, compared with the *Dashboard* page, which provides a summary of participants’ drinking behaviors based upon participants’ interactions with the app:

Well comparing you know forces drinking to others, I’m not sure where that information would have come from.

P15, male

The credibility of InDEx could be enhanced by the presentation of information, in addition to its content. It was suggested that long-term graphs of consumption could capture changes over an extended period, as currently only a 7-day summary graph was available:

I think that (month-long graph) would have been quite good because there are times when you have a heavier drinking week or weekend if you’re away, a family party or whatever. And so the last week isn’t necessarily representative. While at least over a month if you see at least...if you see you’re drinking more heavily over a month, then that is far more than I think than a heavy day or two.

P19, male

#### Theme 2: Meeting Their Needs

Participants consistently emphasized that they used apps on a needs-only basis. In general, the app included a range of features, such as real-time feedback, comparisons with other health behaviors, and goal setting, which seemed to successfully accommodate participants’ varying needs and interests.

Overall, 4 subthemes were developed to represent how InDEx helped to meet participants’ needs, including personalization, internal motivations, being nonjudgmental, and the need for flexibility.

This theme was related to all other themes as the ease of use and credibility contributed to whether participants’ needs were more likely to be fulfilled, which seemed to prompt some change in accountability of their drinking.

##### Personalization

Participants’ preferences varied regarding the frequency of the text messages, the wording used within the app, and the need for InDEx to provide interactive feedback. Most participants reported using pages personalized to their interactions with the app, such as the *Dashboard* and *Add Drink* pages:

Just the comparisons really. (umm) Comparisons for what I’ve had the previous week...Yeah, just...just to see what I’ve had on a weekly basis really.

P21, male

This also extended to the text messages, with participants preferring texts that were personalized through their interactions with the app. Despite personalization being viewed positively, there were mixed responses about the level of personalization within the text messages:

Very informative. Yeah, they were good. (umm) They made...made you think of what you’re putting in your body and what effect it can have and what other solutions you can use. So rather than have a drink, but still be sociable. Yeah, it was good.

P27, male

if you’re filling in..., and you know you haven’t put in your going round to see friends, you know friends places, you’re putting in other information and the system spits out something...‘We’ve noticed you drink mostly at friends houses’ and that’s incorrect. Straightaway it gets your back up.

P22, male

Although personalization was highly regarded, most participants did not want the app or text messages to be further personalized according to their former military occupation:

So I don’t...I don’t sort of look for (umm) I don’t look to be communicated with from anybody in a different way because...what my job has been previously, if that makes sense?

P1, male

...I probably wouldn’t go down the route of...of tying it in with loads of military style brandings and colours and everything. So even...even the language, I probably wouldn’t use military terminology...

P24, male

Participants differed in their opinions of the frequency of text messages, which they felt could be tailored to usage over time or when participants were more likely to drink:

I suppose it just depends on the different...you know different people and what...what suits them. But (umm) but I mean to start with I think daily is fine. And...but then after that I don’t know maybe once or twice a week I suppose.

P4, male

...if I started getting (umm) like Thursday to Sunday when I’m more likely to be drinking, it’ll probably make me more mindful. Whereas if you give them all the time, you start to go yeah that’s another message. Like if you’re only getting them maybe on certain days of the week when you’re likely to drink more...maybe that will make you a bit more mindful of it.

P21, male

The extent to which participants liked the frequency of the messages appeared to contribute to whether they felt InDEx met their needs. In this regard, some participants were dissuaded from engaging with the app on the basis that the frequency was not personalized and therefore applicable or relevant to them:

I was getting texts through the week and to be perfectly honest by the end I was thinking really? You know I’m not out...

P13, male

##### Internal Motivations

Most participants were content with their current drinking habits and therefore engaged with the general monitoring pages of InDEx only, that is, *Add drink* and *Dashboard*. The small proportion describing motivations to change were more likely to use the *Goals* page. Some participants reported becoming more motivated to change their drinking habits over the 28-day period:

Because essentially the App...how ready are you to give up alcohol? (umm) And that was always about an a...you know I’ve always sort of started off as a seven rather than an eight and then I headed towards a nine...

P8, female

Some participants reported that they had previously made significant changes to their alcohol consumption immediately after leaving the Armed Forces or had already set a goal to reduce their alcohol consumption and therefore found behavior change pages, such as the *Goals* page, less useful:

I’ve never thought my drinking was excessive, that I needed to set goals you know (umm) no. So maybe it hadn’t really...it hadn’t really applied to me as much as some others it might.

P9, male

##### Need for Flexibility

Participants expressed a preference for being able to record their alcohol consumption, set goals, and answer questions with little restriction to provide more context to their alcohol consumption. Overall, participants found InDEx relatively nonrestrictive and found some structured features particularly helpful:

Yeah there were enough (drink) options for (umm) which was good rather than having to put...input it yourself. So giving the options of what you had to drink (umm) was easy enough to use.

P9, male

However, some participants felt that drink categories could have been more specific, and drop-down menu options for weekly screening questions allow for open-ended responses so participants could add more contextual details, such as various drinking locations within a drinking session. Some participants suggested that this level of flexibility could improve the accuracy of personalization, particularly regarding the content of text messages:

I’ve gone through everything and for example (umm) it was ‘location you’re having a beer’ (umm) sometimes I wasn’t in any of the five option as in ‘at a friend’s/at a pub/service club/on my own...’

P6, male

I think (umm) one of the...one of the limitations then of actually you know giving your reasons or saying where you were and who you were with...(umm) Because there was a day when I did drink quite a lot, but actually I was at a wedding. But the App didn’t know that.

P26, female

##### Being Nonjudgmental

Participants felt InDEx included positive content and language, and this was particularly important for meeting their needs. In this regard, participants seemed to become disengaged with pages they deemed as judgmental:

Yeah, they were good. Yes (umm) good (umm) advice and it wasn’t too...well it wasn’t patronising or anything, it was...it got back to...to a point.

P24, male

This subtheme relates to *Helpful for ex-serving personnel* as participants seemed more engaged and accepting of InDEx if they felt it was nonjudgmental.

#### Theme 3: Simplicity

Participants believed InDEx was intuitive and easy to use, and this complemented their desire to use apps on a needs-only basis. They preferred to engage with pages of InDEx that did not require too much effort, and its functionality appeared more important than its aesthetics. This also extended to their preference for short and direct text messages. This theme consists of 2 subthemes relating to the app’s clear layout that required minimal effort to use.

##### Clear Layout

Most participants liked the clear layout of the app and felt the presentation of the text and infographics enabled easy interpretation and successful navigation. Participants preferred pages that did not have many hidden features, as these added unwanted complexity and time. Specifically, pages such as the *Dashboard* and *Add drink* were user-friendly and more acceptable to participants than the *Goals* page:

There wasn’t too much information on there, it was all in the page that you needed, you didn’t have to be scrolling around and searching for things and it was there.

P7, female

InDEx seemed to appeal to ex-serving personnel with a range of information technology abilities; however, participants felt they needed a step-by-step tutorial. Although there was an initial navigation page in InDEx, this was not apparent to participants.

So that’s what I did and I think if you go back and look at my data you will see that at some point all of a sudden I went oh hang on, there’s more stuff here! And it was asking me (umm) do I drink alone or with friends or with family, do I drink at home or in a pub? And I didn’t see those...you know I don’t know I didn’t see them, but its...it took me a while to register and go oh I’m missing some information.

P13, male

Although participants generally found the *Add drink* page easy to use, some had difficulty understanding how to add drinks one at a time throughout a drinking session and, as a result, this changed the way they interacted with the app:

So I just remember being a bit confused by [how to add a drink] and thought right I’m not going to try that again.

P1, male

##### Minimal Effort

Participants seemed reluctant to spend too much time engaging with an app and appeared to engage with pages that were not time-consuming. They felt InDEx was user-friendly based on the minimal effort required to record information:

Yeah because...if you can't navigate your way around sometimes it...it makes it difficult so...you know the easier the better really. [InDEx] is user friendly so that’s what you want or what I wanted anyway!

P18, male

Those who set a goal or recorded where and who they were drinking with found this process more complex and found difficulty in interpreting the goals set and amending where and who they were drinking with. This subtheme demonstrates the importance of simplicity and the subsequent impact on user engagement.

#### Theme 4: Helpful for Ex-Serving Personnel

Participants believed InDEx was particularly useful for heavier-drinking ex-serving personnel interested in reducing their alcohol consumption, despite the fact that most participants recruited in this study scored as hazardous drinkers themselves. If perceived as credible, participants felt that they became more accountable for their drinking habits by the end of the study, and this prompted them to make some changes to their alcohol consumption.

This theme therefore related to the helpfulness of InDEx for this population and consists of 3 subthemes, including participants’ accountability for their alcohol consumption, changes in their alcohol consumption, and the app’s usefulness for specific types of ex-serving drinkers.

##### Increased Accountability

Pages, such as the *Dashboard,* were useful for understanding the impact of participants’ alcohol consumption upon aspects of their health and lifestyle by translating their drinks into alcoholic units, calories consumed, and the amount of money spent on alcohol. Many participants reported a lack of knowledge about alcoholic units, and this facility therefore confronted them with the reality of their alcohol consumption:

Eye-opening, you know. All of a sudden it’s in terms of...I’ve had (umm) nine units of alcohol and that’s two burgers, or three burgers or...or that’s an hours walking I’ve got to do! (laugh) Oh! Idiot! So it’s sort of gives you a bit of time to sit back and reflect on.

P10, male

Graphs within the *Drinks* page further supported accountability by participants reviewing consumption over the past week:

I would look up you know how many...usually the drinks sort of tab to see how much I’d been drinking in the last week and think oh I ought...so it would make me look ahead and say well I’m going...if I’m going to need to fit in two or three drink-free days, then...I would use it to say well yeah how many days is it since I’ve had one?...

P19, male

These aspects facilitate participants’ awareness of their alcohol consumption and related risks and increased accountability for their drinking habits.

##### Change of Drinking Habits and Awareness

Some participants reported reductions in their alcohol consumption, which they attributed partially to the information received from InDEx about the impact of their drinking upon their health. Where applicable, goals set by the participant contributed to this, even if they did not meet their goal:

The goal setting was good to get out of that habit. And once you break that habit which you know all is takes you know is that...that week. Then you know you’re onto a good thing because you go what was all the fuss about?

P28, male

Yeah. And did you...how close were you to achieving your goals that you set on the App?

Interviewer

I think I...I folded on the six (laugh) I think I folded on the sixth day!

P8, female

If viewed as credible, the use of comparison information, in particular, appeared to prompt self-reported changes in behavior:

I’d drunk eight beef burgers and I was wondering if I could afford another beef burger!

P28, male

##### Suitability for Types of Drinkers

Participants believed that InDEx was acceptable for ex-serving personnel, particularly for those about to leave the Armed Forces, but questioned their motivations to download the app in the first instance:

(umm) Yeah, as I say I’d recommend [InDEx] to [ex-serving personnel]. It’s whether they take it up.

P17, male

To support uptake, participants felt the app could be advertised within the resettlement package to provide additional support upon leaving service. A resettlement package is given to serving personnel before leaving service and is designed to help prepare them for entering the civilian job market [42].

I think it would be very useful as part of the resettlement package just as a...you know you get a housing brief, you get a finance brief...

P25, male

The app was also regarded as most suitable for heavier drinkers. As demonstrated by their engagement with the *Goals* page, specific features might be appropriate for drinkers incentivized to change their drinking. The following quote is from a participant with an AUDIT score of 10, which is toward the lower end of hazardous alcohol use:

The goals (umm) again it would say ask questions like ‘Do you want to reduce your drinking?’ and realistically no I don’t because I don’t drink that much.

P3, male

Despite drinking at hazardous or harmful levels, none of the participants identified as problem drinkers. The ability for the app to increase a sense of accountability and improve awareness, however, may support the recognition of problems through engagement with InDEx.

## Discussion

### Principal Findings

The main findings of this study indicated that InDEx was an acceptable and user-friendly app for ex-serving personnel to monitor their alcohol consumption. The successes of the app included its personalization, its abilities to meet participants’ needs, the ease of use, and provision of real-time feedback on their drinking. Similarly, the text message facility, in providing more direct communication and personalization, prompted participants to log into InDEx and encouraged engagement. Credibility, relating to whether participants trusted the information provided by the app, was a central facet in determining participants’ level of engagement. Many participants believed InDEx was suitable for heavier drinkers, and although the majority of participants in this study met the criteria for hazardous use, most did not perceive themselves as heavy drinkers.

During the interview, participants reported using self-monitoring pages (*Dashboard* and *Add drink*) the most compared with goal-setting pages (*Goals* and *How you compare)*; this indicates participants’ needs, motivations for using InDEx, and our usability findings. Given that most participants reported that they were not motivated to change their drinking patterns, it is unsurprising that the majority did not set an intentional goal to reduce their drinking and instead engaged with self-monitoring pages of the app. Participants found the app most useful for identifying drinking patterns and understanding the potential impact of their drinking on other aspects of their health. The app may be further developed by incorporating comparison feedback from the *Dashboard* and *Drinks* pages to encourage goal setting. A relevant technique from a previous study used an algorithm that prompted a drinking limit goal via tailored text messaging based on an ecological momentary assessment. These suggested limits were set to be slightly lower than participants’ self-reported alcohol consumption to ensure that the goal was relevant and realistic [43].

Usability findings from our feasibility study suggested a reduction in the number of units of alcohol consumed from week 1 to week 4 of the app study [27]. Our current findings on acceptability suggest self-monitoring and provision of information about participants’ drinking facilitated this change, with many users reporting greater accountability for their own drinking. Self-monitoring, therefore, could be sufficient in prompting a change in drinking behavior without an active plan to change alcohol consumption.

Many participants did not depend on recommended drinking guidelines to identify their unit intake per week, and some participants did not believe the information presented on the *How you compare* page, in particular, comparisons of their drinking with the general population. We used the social norms approach to attempt to address the misperceptions of alcohol consumption in the general population [31]; yet, our sample appeared skeptical. It may be that future developments of InDEx could compare ex-serving personnel’s alcohol consumption with other more relatable populations or include information on the impact of other health behaviors upon alcohol-attributable harms. The latter could be useful for this population as participants reported that the comparisons of alcohol with exercise and food intake were particularly enlightening.

Although personalized pages were deemed more acceptable, participants varied in how personalized they thought InDEx and text messages should be. Most participants preferred personalization based on their interactions with InDEx; for example, providing real-time feedback on their drinking, receiving text messages based upon their common drinks, and tailoring the frequency of the messages to reflect their drinking patterns. Surprisingly, participants indicated a preference for more generic, rather than military-specific, terminology, which may be due to a range of reasons. Previous research suggests that some ex-serving personnel experience problems adjusting to civilian life [5,44]; therefore, it was surprising that there was not a request for InDEx to be framed in the military context, which users may be more comfortable with. It could be that the suitability of military terms may depend on how long someone might have left service.

Although InDEx was acceptable, most participants felt that they would need an explicit incentive to download the app as they did not perceive themselves to be problem drinkers. It is a common perception that individuals believe others have drinking problems rather than themselves [45,46], even if they are drinking at the same level. This may be particularly relevant for the UK Armed Forces, where previous research indicates that those with alcohol problems are the least likely to recognize themselves as having a problem compared with other mental health issues [8,47].

### Comparison With Previous Work

Some of our findings are consistent with previous work, for example, we found that credibility was a central facet of the acceptability of InDEx; Garnett et al’s meta-regression [25] found that the credibility of sources was associated with a reduction in alcohol consumption. Furthermore, the persuasive system design model proposes that perceived system credibility is required for technologies to be believable and more persuasive [48,49]. Previous qualitative research found that information deemed untrustworthy undermined an app’s credibility and users would be less inclined to engage with this information [50]. This may be particularly applicable for our target population as they may not identify themselves as problem drinkers, therefore requiring more persuasion.

We also found that functionality, personalization, and meeting user needs contribute to the acceptability of an app. This is consistent with previous research on the acceptability of smartphone apps in the general population [11,19,41,51]. Participants emphasized needing an app to be easy to use, not to require too much effort, and to contain features providing real-time feedback on their interactions. Contrary to Milward et al’s findings [51], participants seemed to place more emphasis on the need of the app to meet their personal interests and its functionality instead of its aesthetics, preferring simplicity, which seemed to reflect engagement with specific modules [27]. Different preferences may be population specific, as Milward’s study focused on young adults compared with our sample of ex-serving personnel, in which the majority were aged 40 years and older.

Our findings emphasized the need for personalizing both the app and text messages based upon the participant’s interactions with InDEx in terms of usage and when they are likely to drink. This is consistent with previous research conducted in the general population in which the extent to which messages were personalized enhanced engagement and acceptability [23,52,53]. A meta-analysis on the efficacy of SMS text messaging–based interventions showed that message tailoring and personalization were associated with greater intervention efficacy [53]. This suggests that the need for personalization is a common theme across different populations.

One issue highlighted in our study was that participants did not identify themselves as problem drinkers, despite drinking at hazardous levels. This has been found in both military [54] and general populations [55], where individuals understate their drinking and overestimate the drinking of others. This could be explained by the cognitive dissonance theory, whereby ex-serving personnel considered other reasons for why their risk of drinking was higher compared with others, such as their comparators not being truthful about their alcohol consumption or the consumption of food mediating the effect of alcohol [56,57].

Another issue arising from this analysis related to participants’ motivation to change, with many only using the self-monitoring modules, which may reflect that they did not identify having a drinking problem. Research has demonstrated that self-monitoring is associated with greater effect sizes from brief interventions [16] and larger decreases in alcohol consumption and AUDIT scores when using *active ingredients* of BCTs in an alcohol app [58]. Research has previously found motivation to be central to the utilization of smartphone apps and technology [40] and continued engagement for a sustained period of time [19]. More specifically, research on motivation and intention to change drinking behaviors, however, found changes in motivation and self-efficacy predicted drinking outcomes 8 weeks later in the general population [59], but this may differ according to the level of alcohol risk [60]. This indicates that motivation to change may be one component of changing drinking behavior, with other components, such as engagement and self-monitoring, contributing toward this. Therefore, having a self-monitoring module may meet the needs of those who do not identify themselves as problem drinkers and are not motivated to change but may subsequently change their drinking as a result of this module.

There has been limited research on the use of smartphone apps for alcohol misuse for ex-serving personnel; however, 1 study compared these attitudes with smartphone apps for mental health for ex-serving personnel. They found positive overall attitudes to apps as a way of providing strategies to track symptoms [61]. These are similar to our findings where participants believed InDEx was useful for monitoring their consumption; therefore, smartphone apps may be an acceptable delivery method for monitoring drinking behavior in this population.

### Limitations

Participants recruited to the study were selected from a cohort that consented to be contacted for future research and were compensated £40 for taking part; this may have increased engagement with the app. We tried to overcome this by not providing specific instructions on how to use InDEx other than to download it. This may also increase social validity as apps are typically downloaded without additional information [62].

Participants discussed their experience of the InDEx app with a researcher on the InDEx app team, which may have increased the risk of social desirability bias; however, previous research has found that conducting telephone interviews may reduce this risk because of feelings of anonymity [63]. JP also reminded participants throughout the interview that the purpose of the study was to inform intervention development and were encouraged to provide honest feedback. The analysis also involved an independent second coder who also reviewed the codebook and themes.

We also aimed to interview participants up to 1 week after testing the InDEx app, but, in some cases, there was a delay in interviewing participants of over 3 weeks. The researcher generally did not need to prompt participants throughout the interview; however, participants may have had difficulty recalling some pages of the app.

### Conclusions

To the best of our knowledge, this is the first qualitative study to explore the acceptability of a smartphone-based alcohol app for ex-serving personnel. We found that InDEx was an acceptable app for ex-serving personnel to monitor their alcohol consumption. Credibility was a central facet to the acceptability of InDEx and its modules. Pages providing real-time, personalized, and easily interpretable feedback on their drinking and text messages reflecting participants’ interactions with the app were most acceptable.

## Appendix

Multimedia Appendix 1 Copy of topic guide.

Multimedia Appendix 2 Demographic, alcohol use characteristics of participants and number of engagements with InDEx (Information about Drinking in Ex-serving personnel).

## Abbreviations

AUDIT: alcohol use disorder identification test
BCTs: behavior change techniques
InDEx: Information about Drinking in Ex-serving personnel
KCMHR: King’s Centre for Military Health Research
NIHR: National Institute for Health Research
SMS: short message service
