# Supplementary material for: A Qualitative Evaluation of the Acceptability of a Tailored Smartphone Alcohol Intervention for a Military Population: Information About Drinking for Ex-Serving Personnel (InDEx) App
Source: JMIR Mhealth Uhealth. 2019 May 24;7(5):e12267. doi: 10.2196/12267 (PMC6555114; doi:10.2196/12267)
Supplement: Multimedia Appendix 1 [file mhealth_v7i5e12267_app1.pdf]

<u>Appendix 2</u>

Topic guide

Interview topic guide for WP2 – one-to-one qualitative interviews
Participants will be asked if they consent to their quantitative data from the app (including the screening questionnaire) being linked to the qualitative data, which would allow us to triangulate these data.
Introduction:

- Researcher to confirm their name and where they are calling from i.e. Hello, this is Jo-Anne Puddephatt calling from the University of Liverpool.
- Researcher to confirm the name of the participant that they are speaking to i.e. could you please confirm your name?
- Thank them for taking part in the app and for making time to talk about their experience using the app.
- Ask if the participant has any questions about the interview before we start.
- State that the interview will take up to 45 minutes and check that this will be ok.
- Ask if this time is still convenient for the participant.
- Explain that the interview will be recorded with a Dictaphone to ensure we capture all what you say correctly and that their responses will remain anonymous. Ask if this is ok with them.

General questions about the app
Thinking about the app overall…

1. How easy or difficult was it to use?
2. How clear was it to use the app?
3. Would you choose to use it if you weren't part of this study?
    a. If no, how come?
4. Were you able to register and log your responses to questions when you first downloaded the app?
5. Do you think other veterans would want to use it?

    b. If no, how come?

Usage of the app (to be compared with actual usage)
Thinking about how much you used the app…

6. How often did you use the app?
    Prompt: for example, every day, most days, 50% of the days, less than 50% of the days?
7. Did you continue using the app after the month trial?
    a. If yes, how long did you carry on using the app after the month trial?
8. Did you use any particular elements of the app more than others?
    b. For example, the drinks diary?
9. Did you set any goals while using the app?
    c. If yes, how relevant were they to you?
    d. If no, how come? Is there anything we could change on this page to encourage you to use it?

10. Did you read the text messages when they arrived?
11. Where did you tend to use the app? If there is a long pause or ask for clarification:
    e. Prompt: did you use it in a public place or at home?
12. Would you want to carry on using the app?

Usability

Thinking about the different pages of the app…

13. Which elements of the app did you find most useful?
14. What did you think of information you were given after you registered with the app?
    Prompt: for example, the beaker image shown to you
15. How did you find using the drinks diary?
    a. Did you have any problems using it?
    b. Was it quite clear how to log your drinks?
16. How did you find the action planning?
    c. Were the instructions clear?
    d. Relevant to your personal situation?
17. How did you find the content and frequency of the text messages?
    e. Do you think they could be improved? If so, in what way?
18. How easy or difficult was to navigate through the app?
    f. Was it clear to go through different aspects of the app i.e. from the drinks diary to setting goals?
19. Were there any technical problems when you were using the app?

Acceptability

20. What did you think of the appearance of the app?
    a. Are they any improvements that you think could be made?
21. Do you think that the language used was appropriate to veterans?
22. Is there anything that could be changed to make it more suitable to veterans?

    Prompt: i.e. language within the app, or content of the messages?
23. Were you able to fit in using the app with your daily routine?
24. Would you recommend the app to veteran friends or family?
25. Do you have any feedback regarding contact by text message?
    b. Are there other ways you'd prefer to be contacted by the app i.e. push notifications?

Mechanisms of impact

26. Do you think that you changed your drinking consumption or patterns as a result of using the app?
    a. If yes, do you believe that increase or decrease alcohol consumption? How much would you say 20%, around 40% or more than 50%
27. Have your thoughts about alcohol changed at all?
    a. If yes, how?
28. If used goals:
    b. Do you think that any changes in your drinking reflected the goals that you set yourself? If yes, how close to your objectives?

Background

29. How long did you serve in the Armed Forces?
    a. What was your rank while serving?
        i. Non-commissioned Officer, Commissioned Officer or other
    b. Which regiment did you serve in for the longest period of time?
30. Have you used a health app before?
    c. If so, which ones?
31. Have you used an alcohol app before?
    d. If yes, which ones?
32. Have you tried about changing your drinking habits before you started using the InDEx app?

Would you like to add something else about the App?

Closing remarks

Note: Would you like to continue using the InDEx app?

Thank participants for taking part.

Stop recording
