# Supplementary material for: A Qualitative Evaluation of the Acceptability of a Tailored Smartphone Alcohol Intervention for a Military Population: Information About Drinking for Ex-Serving Personnel (InDEx) App
Source: JMIR Mhealth Uhealth. 2019 May 24;7(5):e12267. doi: 10.2196/12267 (PMC6555114; doi:10.2196/12267)
Supplement: Multimedia Appendix 2 [file mhealth_v7i5e12267_app2.pdf]

Appendix 1

Table 1: Demographic, alcohol use characteristics of participants and number of engagements with InDEx.

Participant ID and gender	Age range (years)	Years served in the military	AUDIT score at baseline of InDEx	Total units consumed in week one (median)	Total units consumed in week four (median)	Number of interactions with InDEx
Participant 1, male	40-44	Over 12 years	11	4.54	1.50	21
Participant 2, male	55-64	Over 12 years	11	10.22	7.95	35
Participant 3, male	50-54	Over 12 years	10	7.18	7.66	20
Participant 4, male	25-39	Between 4 and 12 years	12	3.25	6.00	22
Participant 5, male	55-64	Over 12 years	7	3.59	3.19	35
Participant 6, male	40-44	Over 12 years	11	12.00	2.00	16
Participant 7, female	40-44	Less than 4 years	12	9.75	6.81	20
Participant 8, female	40-44	Over 12 years	12	9.75	4.41	55
Participant 9, male	45-49	Over 12 years	8	10.08	6.00	38
Participant 10, male	50-54	Over 12 years	12	4.40	3.50	31
Participant 11, male	45-49	Over 12 years	10	7.19	6.27	39
Participant 12, male	40-44	Over 12 years	11	4.54	1.50	21
Participant 13, male	50-54	Over 12 years	16	9.39	3.14	29
Participant 14, male	55-64	Over 12 years	10	11.99	5.00	34
Participant 15, male	50-54	Over 12 years	10	8.00	6.00	11

Participant 16, male	25-39	Between 4 and 12 years	15	9.50	7.20	53
Participant 17, male	50-54	Over 12 years	12	16.00	5.74	42
Participant 18, male	45-49	Over 12 years	10	4.77	6.63	18
Participant 19, male	55-64	Over 12 years	11	5.68	2.74	50
Participant 20, male	55-64	Over 12 years	7	3.59	3.19	35
Participant 21, male	40-44	Over 12 years	14	6.81	9.00	45
Participant 22, male	45-49	Over 12 years	10	2.16	1.00	49
Participant 23, male	25-39	Over 12 years	14	13.92	6.17	28
Participant 24, male	25-39	Between 4 and 12 years	10	4.54	9.19	48
Participant 25, male	50-54	Over 12 years	13	3.07	4.37	34
Participant 26, female	25-39	Between 4 and 12 years	5	3.89	1.62	29
Participant 27, male	55-64	Over 12 years	9	14.00	1.00	35
Participant 28, male	55-64	Over 12 years	14	5.86	3.13	46
Participant 29, male	55-64	Over 12 years	12	5.68	4.32	40
